# Malignant upper urinary tract obstruction in cancer patients: A systematic review

**DOI:** 10.1002/bco2.340

**Published:** 2024-02-27

**Authors:** Max Shah, Francesca Blest, James Blackmur, Alexander Laird, Shoba Dawson, Jonathan Aning

**Affiliations:** ^1^ University of Bristol Bristol UK; ^2^ Department of Urology Cambridge University Hospitals NHS Foundation Trust Cambridge UK; ^3^ Early Cancer Institute University of Cambridge Cambridge UK; ^4^ Department of Urology, Western General Hospital Edinburgh UK; ^5^ Institute of Genetics and Cancer The University of Edinburgh Edinburgh UK; ^6^ Bristol Urological Institute, Southmead Hospital North Bristol Trust Bristol UK; ^7^ Population Health Sciences, Bristol Medical School University of Bristol Bristol UK

**Keywords:** malignant ureteral obstruction, nephrostomy, reporting framework, ureteric stent, urinary diversion

## Abstract

**Objective:**

To systematically summarise the current clinical evidence for de novo malignant upper urinary tract obstruction treatment with a focus on standards of reporting, patient outcomes and future research needs.

**Methods:**

This review protocol was published via PROSPERO (CRD42022341588). OVID MEDLINE (R), EMBASE, Cochrane Central Register of Controlled Trials—CENTRAL were searched up to June 2022 in accordance with the Preferred Reporting Items for Systematic Reviews and Meta‐analyses. Prospective and retrospective studies were included.

**Results:**

Of 941 articles identified, 82 with 8796 patients were eligible for inclusion.

Most studies in the published literature are retrospective and investigate heterogenous malignancies. Percutaneous nephrostomy and ureteric stenting are the most studied interventions. Few studies describe the outcomes from no intervention or investigate patient perspectives. Overall reported median survival after intervention was around 11.7 months. A lack of standardised reporting of outcomes was evident.

**Conclusions:**

Malignant upper urinary tract obstruction is an important clinical condition affecting patients globally. Overall survival after intervention appears poor however the current evidence base has significant limitations due to studies of low methodological quality and the lack of a standardised framework for reporting outcomes.

We have provided a pragmatic framework for future studies based on the review to ensure a uniform methodology is utilised moving forward.

## 1 INTRODUCTION

Malignant upper urinary tract obstruction (MUUTO) in cancer patients may be caused by intrinsic, intramural or extrinsic compression from malignant disease. The incidence of MUUTO is presently unknown; however, the development of MUUTO is commonly, rightly or wrongly, regarded as being associated with a poor prognosis. MUUTO may affect patients with a wide range of cancers, and treatment may be offered to decompress MUUTO to improve symptoms and extend overall survival.

Patients with MUUTO often have associated significant morbidity and the benefit of intervention to decompress the obstruction can be uncertain. Little detailed clinical guidance or information is available to inform the management of patients with MUUTO or inform counselling and shared decision‐making.

Recent systematic reviews have focussed on assessing ureteral stent and percutaneous nephrostomy (PCN) efficacy and outcomes in heterogeneous populations.^1^, ^2^, ^3^ The outcomes of no intervention have not been examined systematically. Additionally, although the limitations of systematic reviews to date have been attributed to their inclusion of mostly retrospective studies; no in‐depth review of the methodologies used in published papers to date has been performed. There is an urgent need to define standards for future MUUTO studies in order to ensure that patient care in this important field improves. The objective of the present study was to systematically review the evidence for MUUTO treatment and outcomes, with a focus on reporting standards, in order to inform the development of robust methodology for future research.

## 2 METHODS

### 2.1 Evidence acquisition

#### 2.1.1 Search strategy and screening

This study was prospectively registered on the PROSPERO International Prospective Register of Systematic Reviews (CRD42022341588). The review was conducted in concordance with the recommendations defined in the Preferred Reporting Items for Systematic Reviews and Meta‐Analyses statement. Table 1 summarises the study Population, Intervention, Comparator, Outcome, Study design (PICOS).

**TABLE 1:** Population, Intervention, Comparator, Outcomes, Study design (PICOS) for systematic review.

	Inclusion	Exclusion
**P**opulation	Persons aged ≥18 years with de novo malignant obstruction of the upper urinary tract caused by cancer.	Studies with datasets that included mixed benign and malignant causes of upper urinary tract obstruction.
**I**ntervention	Treatment of de novo malignant obstruction of the upper urinary tract: ureteric stent, percutaneous nephrostomy, urinary diversion procedure, ureteric reimplantation, no intervention	Studies with datasets that did not separate outcome data by intervention
**C**omparator	Not applicable	
**O**utcomes	Effect of intervention on renal function, survival, complications, inpatient length of hospital stay at presentation, readmission rates, quality of life, prognostic models	Studies without survival outcome data
**S**tudy design	Quantitative and qualitative observational and interventional studies	Case studies, unpublished studies, letters, reviews, conference abstracts and case series with less than five patients.

The search strategy included the following databases: OVID MEDLINE (R) (1946–June 2022), EMBASE (1974–June 2022), Cochrane Central Register of Controlled Trials—CENTRAL (in The Cochrane Library—2022). The complete search strategy is attached as Supplementary File 1.

One author (MS) carried out the search. Two authors (MS and FB) screened 50% of the titles and abstracts each to exclude clearly irrelevant papers; 100% of these were then verified in conjunction with a senior author (JA). Full texts were retrieved if papers could not be excluded based solely on title and abstract. All full texts were screened independently by two authors (MS and FB). In 20% of the full texts screened, there were conflicts or papers for which it was unclear whether the inclusion criteria were satisfied. These were discussed with a senior author (JA) until a consensus was achieved.

#### 2.1.2 Inclusion criteria

Inclusion criteria were English language manuscripts reporting on interventions for de novo malignant ureteric obstruction in adult patients. We included observational and interventional studies. Case studies, unpublished studies, letters, reviews, conference abstracts and case series with less than five patients were excluded. Papers including reports on benign diseases were only included if survival data on de novo MUUTO cases could be extracted separately; otherwise, they were excluded as ‘mixed population’. Similarly, those that investigated multiple intervention types but reported survival data that was not separated by intervention were excluded as ‘mixed intervention’. Studies were also not included if they contained no data on survival.

#### 2.1.3 Data extraction

Two authors (MS and FB) extracted data independently for 50% of the papers each. A senior author (JA) then extracted data from 10% of the papers independently and checked equivalence to ensure accuracy of extractions. A standardised form was developed to record the study design and study outcomes (including survival, quality of life [QOL], complications, effect on renal function, readmission, receipt of additional treatment after intervention and prognostic models). Meta‐analyses were planned but were deemed inappropriate due to data heterogeneity.

## 3 RESULTS

### 3.1 Evidence synthesis

**FIGURE 1:**
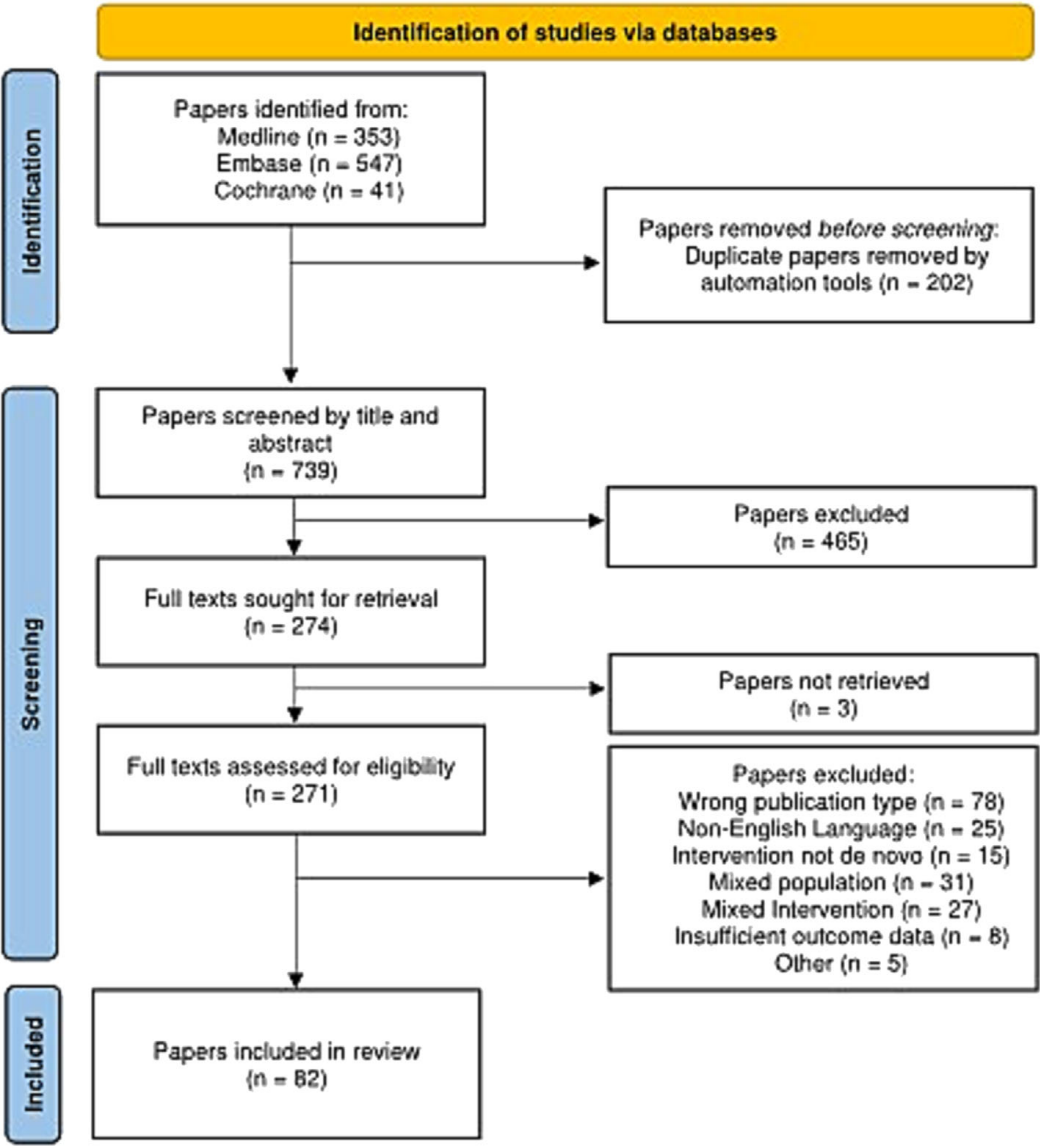
PRISMA flow diagram of evidence acquisition in a systematic review of malignant upper urinary tract obstruction.

After duplicates were removed, the search identified 739 papers. Of these, 468 were excluded by title and abstract screening, and a further 189 were excluded after full‐text assessment. Of the studies screened, 82 met the study criteria and were included in this review.^4^, ^5^, ^6^, ^7^, ^8^, ^9^, ^10^, ^11^, ^12^, ^13^, ^14^, ^15^, ^16^, ^17^, ^18^, ^19^, ^20^, ^21^, ^22^, ^23^, ^24^, ^25^, ^26^, ^27^, ^28^, ^29^, ^30^, ^31^, ^32^, ^33^, ^34^, ^35^, ^36^, ^37^, ^38^, ^39^, ^40^, ^41^, ^42^, ^43^, ^44^, ^45^, ^46^, ^47^, ^48^, ^49^, ^50^, ^51^, ^52^, ^53^, ^54^, ^55^, ^56^, ^57^, ^58^, ^59^, ^60^, ^61^, ^62^, ^63^, ^64^, ^65^, ^66^, ^67^, ^68^, ^69^, ^70^, ^71^, ^72^, ^73^, ^74^, ^75^, ^76^, ^77^, ^78^, ^79^, ^80^, ^81^, ^82^, ^83^, ^84^, ^85^ Figure 1 illustrates the process. Of the five papers excluded for ‘other’, three were yet to be published, and two were duplicates.

#### 3.1.1 Study design and characteristics

Of the 82 studies included, most described the outcomes of intervention utilising either or both PCN and ureteric stenting (*n* = 82) to treat MUUTO. The outcomes of polymeric stents (*n* = 20 including 1749 patients), metallic stents (*n* = 9 including 292 patients) and tandem stents (*n* = 1 including 34 patients) were evaluated in specific studies. Only a small number of studies included surgical urinary diversion (*n* = 6 including 188 patients) or no intervention (*n* = 5 including 266 patients).

MUUTO studies in the published literature were conducted across six continents; all were conducted in secondary care, and most (*n* = 78 including 5590 patients) were single‐centre studies.

Of the papers reviewed 74 were retrospective, six were prospective, and only two were randomised controlled trials. Javanmard et al.^4^ randomised 86 prostate cancer patients with MUUTO to either ureteral reimplantation or PCN and evaluated survival, renal function and complications. Although both methods of diversion achieved similar mean decreases in creatinine, the mean survival rate was significantly higher in the ureteric implantation group 22.42+/−0.87 months versus the PCN group 20.48+/−0.65 months (*p* = 0.0001). They also found that 100% of the patients in the nephrostomy placement group experienced complications and social inconvenience; a lower proportion of patients suffered complications and perceived inconvenience in the re‐implantation group. The authors acknowledged that their study may be biased because healthier patients may have been more likely to be fit to undergo reimplantation. Kim et al.^5^ randomised 19 patients with MUUTO caused by Stomach, Colorectal or Gynaecological cancer to either a covered metal stent arm or a double‐J polymeric stent arm and recorded survival, technical success of procedure, complications and patency rates. They found that there was no difference in overall survival rates between the tested interventions, but overall patency rates were higher in the covered metal stent group. The sample size in both studies described was small limiting their conclusions. Additionally, when assessed for the primary outcome of survival, using the Cochrane risk of bias 2 tool ‘some concern’ was demonstrated for both studies due to a lack of clarity in the randomisation process. Table 2 summarises the characteristics of the included studies.

**TABLE 2:** Characteristics of the included studies.

Characteristic	Values from included studies
Year of publication	
Pre‐1990	12 (15%)
1990–1999	18 (22%)
2000–2009	15 (18%)
2010–2022	37 (45%)
Location	
Asia	37 (45%)
North America	20 (24%)
Europe	19 (23%)
Australasia	3 (4%)
South America	2 (2%)
Africa	1 (1%)
Number of centres in study	
Single centre	78 (95%)
Multicentre	4 (5%)
Study type	
Retrospective:	74 (90%)
Single cancer	19
Mixed	55
Prospective non‐randomised:	6 (7%)
Single cancer	4
Mixed	2
Randomised controlled trials:	2 (2%)
Single cancer	1
Mixed	1
Intervention studied	
PCN alone	35 (43%)
Stent alone	17 (21%)
Both PCN and stent	19 (23%)
Urinary diversion and either stent or PCN or both	6 (7%)
No intervention and PCN +/− stent	5 (6%)
Primary outcome measure	
Survival	58 (71%)
Stent patency	22 (27%)
Complications	2 (2%)

Abbreviation: PCN, percutaneous nephrostomy.

#### 3.1.2 Study population

In total, 8796 (range 7–2958) MUUTO patients were studied, where (*n* = 78 studies) 3334/8567 (39%) were female. The most prevalent primary malignancies studied causing MUUTO were prostate cancer and cervical cancer in men and women respectively. Less than half of the studies clearly defined the metastatic or locally advanced cancer status of those included: 30 studies defined the study population as including both metastatic and non‐metastatic patients, two studies included metastatic patients only, and the remaining 50 studies did not clearly define the studied population. Only two papers stated the ethnicity of participants: the first studying a cohort with gynaecological malignancies where 68.3% were African American, 19% were Caucasian and 12.7% were described as other.^6^ In the other paper studying a cohort of patients with prostate cancer 80% were White, 13% were Black, 3% were Hispanic and 5% described as other.^7^ Although limited these studies suggest that MUUTO affects all ethnicities. Table 3 illustrates in detail the characteristics of the patients included in studies.

**TABLE 3:** Characteristics of patients investigated by the included studies.

Variables (number of papers that recorded this data)	Values from included studies	
Sex (78/82)		
Male	61% (5226)	
Female	39% (3334)	
Aggregate mean age (55/82)	65.5	
Mean lower–upper limit of age (52/82)	32–82	
Median age (19/82)	60	
**Malignant primary**	**No. of patients**	**Percentage**
Prostate	3651	41.41
Cervix	1589	18.02
Colorectal	650	7.37
Bladder	553	6.27
Gastric	502	5.69
Gynaecological	427	4.84
Gastrointestinal	299	3.39
Uterus (endometrial)	207	2.35
Ovarian	206	2.34
Genitourinary	164	1.86
Other	111	1.26
Breast	105	1.19
Lymphoma/sarcoma	94	1.07
Urothelial	53	0.60
Lung	50	0.57
Gallbladder	32	0.36
Retroperitoneal	25	0.28
Pancreas	20	0.23
Metastatic (primary not given)	17	0.19
Haematological	12	0.14
Unknown origin	11	0.12
Vulval	7	0.08
Renal	7	0.08
Testicular	7	0.08
Oesophageal	6	0.07
Osteologic	4	0.05
Choriocarcinoma	2	0.02
Head and neck	2	0.02
Melanoma	2	0.02
Neuroblastoma	1	0.01

#### 3.1.3 Outcomes of intervention

The primary clinical aims of intervention to decompress MUUTO are to improve renal function, QOL and overall survival. A lack of standardisation in the reporting of these outcomes in the included studies was evident.

##### Renal function change and receipt of additional cancer therapy after MUUTO treatment

Around half of the studies (*n* = 42 including 1188 patients) examining interventions for MUUTO described the resulting effect of treatment on renal function. The specific time before and after intervention that renal function was measured was rarely reported and where this was defined, it was variable. Renal function was measured from admission to immediate pre‐intervention and from immediate post‐intervention to 12 months post‐intervention. Although all 42 studies described an overall improvement in renal function after intervention, only four studies reported the change in renal function on a per‐patient basis. These were largely historical papers with small sample sizes, but they did demonstrate that not all patients' renal function will improve with intervention. Changes in serum creatinine were most frequently measured (*n* = 25), in 1188 patients a mean pre‐intervention value of 4.72 mg/dL decreased to 1.62 mg/dL post‐intervention. Just under half the papers (*n* = 38) recorded if an intervention was performed on one renal unit or two simultaneously, with a total of 1580 and 831 patients, respectively. Of these, only four studies of 124 patients separated renal function data by whether patients received unilateral or bilateral intervention,^8^, ^9^, ^10^, ^11^ with the rest not splitting the data or splitting by cancer type or type of intervention given. Bilateral interventions were reported to confer a greater decrease in mean serum creatinine concentrations with a drop of 5.21 versus 3.5 mg/dL in unilateral interventions. However, the mean pre‐intervention concentration was also higher in the bilateral patients, which skews this comparison; mean pre‐ to post‐intervention serum creatinine of bilateral and unilateral patients, respectively, were 6.79 to 1.58 mg/dL in 102 patients and 5.35 to 1.85 mg/dL in 22 patients.

Only 26 studies documented receipt of additional cancer treatment after MUUTO decompression. Treatments were received by 792/1234 (64%) patients in these studies and included radiotherapy, chemotherapy, hormonal therapies, immunotherapies, surgery and/or a combination of these. Only three papers (including 186 patients) specified the intent of the therapy, of which 108 patients received palliative treatment and 26 treatment with curative intent.^12^, ^13^, ^14^ Only 14 papers of 496 patients documented both improvement in renal function and receipt of additional cancer therapy.

##### Overall survival after MUUTO treatment

There was variation in how survival after MUUTO treatment was reported in studies. Some presented survival after MUUTO treatment as median months survived, whereas others used a mean. Some studies described survival as the percentage of patients still alive at a time point after intervention ranging from 6 months to 10 years. One study just stated that three patients died after intervention with no further information.^15^

Survival after intervention was generally not classified by the presence or absence of metastatic disease. Although some papers examined factors associated with survival, none specifically presented Kaplan–Meier survival curves demonstrating whether cancer stage or past or future treatment affected overall survival. These are major limitations to the interpretation of survival data reported after intervention for MUUTO. Manuscripts reported overall survival after intervention as a mean or a median of included patients. The weighted average of these means was 9.6 months (*n* = 22 including 1267 patients), and of medians was 11.7 months (*n* = 42 including 6363 patients). Average median survival for those who underwent no intervention was 4.2 months (*n* = 5 studies including 149 patients). Details of the weighted survival data in months are shown in Table 4. Survival by intervention type showed that stented patients were reported to live longer than those who received nephrostomy. In the five papers that reported metal stent‐only survival data, the average survival was 6.1 months in 276 patients, whereas across 10 papers polymeric‐only stents averaged a survival of 8.2 months. Only three papers documented separated survival data for surgical diversion and this averaged 19.1 months in 71 patients. Papers did not split survival data by those that had metastatic disease and those that had further adjuvant treatment for their cancer and as such the overall survival data draw from a heterogenous population, at high risk of selection bias.

**TABLE 4:** Survival data.

Survival (number of papers that reported this)	Months (number of patients)
Overall survival	
Median (42)	11.7 (6363)
Mean (22)	9.6 (1267)
Median survival by intervention	
PCN (18)	6.3 (1467)
Stent (13)	8.3 (1003)
Median survival by malignancy type	
Prostate (7)	18.8 (170)
Bladder (7)	11.7 (144)
Cervical (6)	15.9 (117)
Colorectal (5)	7.7 (91)
Gastric (2)	4.2 (37)

Abbreviation: PCN, percutaneous nephrostomy.

In the 38 papers, which recorded if an intervention for MUUTO was performed on one renal unit or two simultaneously, survival after decompression of one renal unit versus two renal units was described by only Nariculam et al. in 25 patients (unilateral: 3 months in seven patients; bilateral: 9.3 months in 18 patients).^11^ There were six papers that investigated laterality as a predictor of survival with regression analysis; none of these papers found it to be significant in univariate analysis.^8^, ^13^, ^16^, ^17^, ^18^, ^19^ However, Izumi et al. use laterality in their validated multivariate Primary site, Laterality, serum Creatinine level, and Treatment for primary site (‘PLaCT’) score, which is a predictor for survival.^16^

Survival by specific malignancy type was reported in 12 studies, and of these, prostate cancer and cervical cancer patients had the greatest median survival, but the numbers of patients included were limited. For the full table of survival data by cancer type description see Supplementary File 2.

##### Complications

Data about complications resulting from intervention are vital to understanding the morbidity of decompression procedures and were reported in 61 studies. There was little consistency in the reporting of complications. More comprehensive studies reported the number of participants who experienced each complication, whereas others reported only a percentage of the participants. Urinary tract infection and dislodgement of nephrostomy tube both affected around one‐quarter of patients undergoing treatment, although the timing of the suffered complication was often not reported. Of 19 studies investigating both nephrostomy and stent, 10 did not separate complication data, three did not report complications,^7^, ^20^, ^86^ one reported only ‘incidences’ of complications^21^ and five reported complications split by intervention.^22^, ^23^, ^24^, ^25^, ^26^ None of the studies used the Clavien–Dindo classification system and as such it was difficult to ascertain the seriousness of complications reported. The complications with the highest incidences in those receiving PCN were urinary tract infection (UTI) (30% of 565 patients), haematuria (26% of 595 patients) and dislodgement of nephrostomy tube (24% of 717 patients), whereas among those that received a stent, they were UTI (21% of 207 patients), stent failure (18% of 597 patients) and haematuria (7% of 480 patients). Table 5 illustrates the complications defined. Of note, we were not able to define whether stent failure was more common in certain malignancies as no studies stratified complication data in this way.

**TABLE 5:** Complications in descending order of number of patients affected.

Complications *n* = 61/82	Number of studies that reported this complication	Total number of patients in the studies that reported a complication	Number of patients affected (% of total patients in those studies)
Urinary tract infection	27	1829	431 (24%)
Dislodgement of nephrostomy tube	24	1393	340 (24%)
Haematuria	21	1638	251 (15%)
Stent failure	17	1267	226 (18%)
Obstruction of nephrostomy tube	18	996	178 (18%)
Pain	14	1096	131 (12%)
Sepsis	13	722	75 (10%)
Other urinary symptoms	5	407	67 (16%)
Skin infection	7	530	61 (12%)
Dislocation of stent	7	317	22 (7%)
Haemorrhage^a^	9	458	18 (4%)
Bladder irritation	6	258	18 (7%)
Other^b^	6	472	53 (11%)

^a^ Includes haemorrhage and haemorrhage requiring transfusion.

^b^ Includes nausea and vomiting, electrolyte disturbance, hypotension, generic cardiovascular symptoms, generic gastrointestinal symptoms, pulmonary embolus, anastomotic stricture and small bowel obstruction.

##### Time spent in hospital peri‐intervention

Peri‐intervention inpatient length of stay for patients admitted with MUUTO was reported by 10 studies, comprising 523 patients. Inpatient length of stay in these studies was on average 21 days. In total, 16 studies documented the number of patients who died in hospital during their admission to MUUTO. Of 694 patients in these studies who received an intervention for MUUTO, either ureteric stent or PCN, 18% died on the same inpatient admission. Although it is clear that a significant number of patients may die on the same inpatient admission after intervention, the absolute proportion is not clear from our review of the literature because these data are not clearly reported in most studies.

##### Readmission rates

Only five papers stated how many patients were readmitted to hospital after MUUTO treatment.^27^, ^28^, ^29^, ^30^, ^87^ Readmission rates were high in these studies with a reported 304/344 (88%) patients readmitted. However, these papers did not explicitly state whether the readmissions were a direct result of a complication of the intervention to treat MUUTO. It is noteworthy that although many studies recorded complications of interventions (*n* = 61) most failed to document whether readmission was associated with the complication. This is an area for improvement in future studies.

##### Quality of life

The effect of treatment on improvement in QOL in patients with MUUTO was measured or commented on in 22 studies. There was little standardisation of QOL assessments with 15 studies making generalised comments about how much of the patient's remaining life they spent in hospital and three studies using unspecified QOL scoring systems.^31^, ^32^, ^33^ One of these three used their unspecified QOL score alongside the international prostate symptom score and overactive bladder symptoms score.^33^ One paper used the Functional Assessment of Cancer Therapy—General (FACT‐G) score, which is intended to measure general QOL in patients undergoing cancer therapy.^34^ This paper compared QOL in patients who received either nephrostomy or stenting and those who required diversion but did not receive it. Although survival was longer in the intervention group, there was no difference in QOL scores. In fact, no papers reported a significant improvement in QOL after intervention. The remaining three papers assessed QOL using the Grabstald and McPhee QOL criteria.^35^, ^36^, ^37^ They found that 47/75 (63%) participants across the three studies would fulfil the criteria defined as ‘a life in which the patient has minimal pain, few complications, full mental capacity, few complications relating to PCN insertion and the ability to return home for at least two months prior to death to participate in family life’. These data suggest that a significant minority (37%) perceive that they do not have a useful QOL after intervention, and this merits further investigation. Additionally, the Grabstald and McPhee criteria for nephrostomy and cancer patients were defined in 1973. There is a need to evaluate qualitatively whether these factors alone remain important to contemporary patients with MUUTO or whether additional factors should be considered.

##### Predictive modelling to determine MUUTO outcomes

A number of studies (*n* = 26) investigated whether prognostic markers could be determined for MUUTO treatment outcomes in their cohort. The primary outcome in these studies was survival. A wide array of variables inclusive but not exhaustive of hypoalbuminaemia, hyponatraemia, type and stage of malignancy, degree of hydronephrosis, degree of renal impairment, functional status and age. Although many studies looked retrospectively at the impact of whether patients went on to receive further treatment, none that the authors could find examined whether further treatment was intended or planned at the time of MUUTO intervention. Of note hypoalbuminaemia, severely raised pre‐treatment creatinine levels and degree of hydronephrosis were identified as poor prognostic indicators in *n* = 6/26 studies. One paper by Ishioka et al.^38^ proposed a model that has been validated and adapted by four other studies.^17^, ^30^, ^39^, ^40^ Hypoalbuminaemia is the only prognostic factor common to all of these papers. Two further papers by Izumi et al.^16^ and Cordeiro et al.^88^ have proposed models that have been validated by one other paper each.^89^, ^90^ Izumi et al. validated their own ‘PLaCT’ score for primary cancer. The model by Cordeiro et al. was validated for use in cervical cancer patients utilising their Eastern Cooperative Oncology Group (ECOG) status and a number of events related to malignancy.

#### 3.1.4 Proposals for a best practice framework for future study design and future research

The findings of this review have clearly identified the limitations of the present literature and the inconsistency in reporting outcomes from MUUTO treatment, which precludes meta‐analysis. There are currently no recommendations for standards of MUUTO reporting. We have therefore developed a dedicated good practice framework derived from the evidence, **M**alignant upp**E**r uri**N**ary **T**ract **O**bstruction f**R**amework (MENTOR), to fill this void.

##### MENTOR recommendations

We recommend that MUUTO studies must be designed to provide clinically relevant patient population‐specific data. As such, we recommend that the following essential criteria be included in manuscripts describing MUUTO treatment.

Definition of MUUTO population

- In the demographic information as a minimum standard ethnicity, sex and primary malignancy of patients included should be stated.
- The patients should be defined as having either MUUTO with or without metastatic disease.
- The mode of presentation of patients should be stated as either elective or emergency. It should be made clear whether patient group studied are intervention naïve for MUUTO.
- The environment in which the patients are studied should be clearly stated as either in the community or in secondary care.

Study methodology

- Intervention studied should be clearly defined. Where no intervention is studied, this should be clearly described.
- The primary outcome should be clearly stated.
- Renal function change after intervention should be documented. In principle, the defined measurement point pre‐intervention should be the lowest renal function measurement prior to intervention and after intervention, the defined point post‐intervention should be the last renal function measurement prior to discharge from hospital.
- Complications after intervention should be recorded using standardised nomenclature (such as Clavien Dindo).
- Readmissions after intervention should be documented and the reason for readmission clearly described.
- A clear description of median overall survival in months should be presented, stratified by cancer stage and past and future receipt of treatment for their cancer.

**FIGURE 2:**
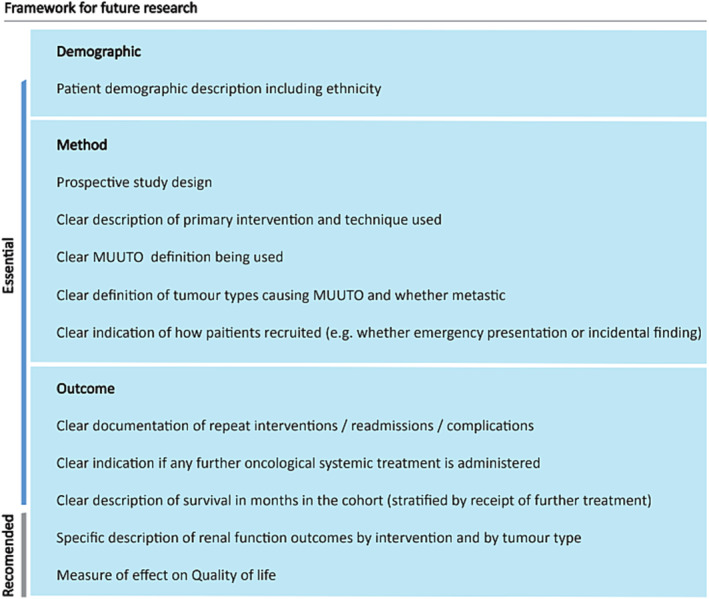
Framework for future research. MUUTO, malignant upper urinary tract obstruction.

It is desirable that future studies would be prospective, details regarding treatment received after intervention are recorded and data collected on QOL after management utilising validated questionnaires. Figure 2 illustrates the framework proposed.

##### Recommendations for future research

To our knowledge, this is the most comprehensive contemporary systematic review of MUUTO treatment. We provide a clear overview of current research in this area, but more importantly, we highlight the limitations in the present literature. Although there is a need for consensus guidelines in the management of this patient group, which this group will seek to address by means of a Delphi consensus, there are pressing research needs in this area of urological care.

There is an urgent need to define the prevalence of MUUTO, to establish the true burden of this condition on the health system, particularly as it must be recognised that not all patients with MUUTO will be referred for intervention. There is a need to better understand the benefit of intervention in the cohort of patients that present to hospital as an emergency and whether these patients who are acutely unwell should be managed differently to cases discovered incidentally. Research to look at the difference in outcomes between immediate decompression in comparison to deferred treatment will be valuable in this space. It is unknown whether survival outcomes are different depending upon the primary tumour causing the malignant obstruction and investigating specific populations can answer this question. Although multiple studies have investigated predictive models, there is a need to validate these tools before their widespread use can be incorporated into clinical practice.

Finally, there is a lack of information on the impact of interventions to relieve obstruction on patient's QOL. Further research into patients' priorities and preferences, and additional qualitative research into the effects of intervention versus no intervention on QOL and survival is desperately needed.

Addressing all these gaps in knowledge will help progress the field so that we can properly enable shared decision making in this complex clinical situation.

## 4 CONCLUSIONS

Malignant upper urinary tract obstruction is a urological condition that affects patients globally and has significant unmet needs. The best treatment method is unknown. Intervention to decompress the upper tract seems to achieve an improvement in renal function but there is a variation in the effect of intervention on overall survival and many patients do not return to a good QOL after intervention. Through an extensive literature review, we have recommended the adoption of the MENTOR framework in future studies which sets realistic standards for the presentation of MUUTO study results and also proposed recommendations for future research.

## AUTHOR CONTRIBUTIONS

Jonathan Aning had full access to all the data in the study and took full responsibility for the integrity of the data and the accuracy of the data analysis. *Study concept and design:* Aning, Dawson. *Acquisition of data:* Shah, Blest, Dawson. *Analysis and interpretation of data:* Shah, Blest, Aning. *Drafting of the manuscript:* Shah, Blest, Aning. *Critical revision of the manuscript for important intellectual content:* Blackmur, Laird. *Obtaining funding:* None. *Supervision:* Aning. *Other:* None.

## CONFLICT OF INTEREST STATEMENT

The authors declare that they have no disclosures or conflicts of interest.

## Supporting information


**Data S1.** Supplementary File 1: Search strategy
Supplementary File 2: Full table of survival by cancer type
Supplementary File 3: Table of included papers
