# Supplementary material for: Malignant upper urinary tract obstruction in cancer patients: A systematic review
Source: BJUI Compass. 2024 Feb 27;5(5):405–16. doi: 10.1002/bco2.340 (PMC11090775; doi:10.1002/bco2.340)
Supplement: Supplementary file 1 — Data S1. Supplementary File 1: Search strategy Supplementary File 2: Full table of survival by cancer type Supplementary File 3: Table of included papers [file BCO2-5-405-s001.docx]

**Supplementary material**

**Supplementary File 1: Search strategy**

| MEDLINE |
| --- |
| Adults with malignant ureteric obstruction (Population) |
| Hydronephrosis/ or Ureteral Obstruction/ or Ureter/ or ureter* obstruction.mp.  (Cancer or malignan*).ti.  1 and 2 |
| AND |
| Decompression by PCN, ureteric stent, diversion, or no treatment (Intervention) |
| Decompression/ or Decompression, Surgical/ or Decompression.ti.  Stents/ or ureteric stent.ti.  Nephrostomy, percutaneous/ or (percutaneous nephrostomy or nephrostomy or PCN).ti.  urinary diversion.ti. or exp Urinary Diversion/ or ureter* reimplantation.mp.  Or/4-7  3 and 8 |
| Results = 353 |

| EMBASE |
| --- |
| Adults with malignant ureteric obstruction (Population) |
| Hydronephrosis/ or Ureteral Obstruction/ or Ureter/ or ureter* obstruction.mp.  (Cancer or malignan*).ti.  1 and 2 |
| AND |
| Decompression by PCN, ureteric stent, diversion, or no treatment (Intervention) |
| Decompression/ or Decompression, Surgical/ or Decompression.ti.  Stents/ or ureteric stent.ti.  Nephrostomy, percutaneous/ or (percutaneous nephrostomy or nephrostomy or PCN).ti.  urinary diversion.ti. or exp Urinary Diversion/ or ureter* reimplantation.mp.  Or/4-7  3 and 8 |
| Results = 547 |

Cochrane:

Date Run: 21/06/2022 00:10:24

ID Search Hits

#1 MeSH descriptor: [Hydronephrosis] explode all trees 64

#2 MeSH descriptor: [Ureteral Obstruction] explode all trees 109

#3 MeSH descriptor: [Ureter] explode all trees 208

#4 (ureter* obstruct*):ti,ab,kw (Word variations have been searched) 523

#5 #1 or #2 or #3 or #4 719

#6 cancer or malignan*:kw,ti,ab (Word variations have been searched) 214611

#7 MeSH descriptor: [Neoplasms] explode all trees 88476

#8 #6 or #7 231888

#9 #5 and #8 109

#10 MeSH descriptor: [Decompression] explode all trees 194

#11 MeSH descriptor: [Decompression, Surgical] explode all trees 1222

#12 (decompression):kw,ti,ab (Word variations have been searched) 3656

#13 #10 or #11 or #12 4393

#14 MeSH descriptor: [Stents] explode all trees 4538

#15 (ureteric stent*):kw,ti,ab (Word variations have been searched) 902

#16 #14 or #15 5249

#17 (ureter* implant*):ti,kw,ab (Word variations have been searched) 62

#18 MeSH descriptor: [Nephrostomy, Percutaneous] explode all trees 234

#19 (percutaneous nephrostomy or nephrostomy or PCN):kw,ti,ab (Word variations have been searched) 613

#20 urinary diversion:kw,ti,ab (Word variations have been searched) 570

#21 MeSH descriptor: [Urinary Diversion] explode all trees 338

#22 [OR #17-#21] 1239

#23 #13 or #16 or #22 10694

#24 #9 and #23 41

Searches

Medline 353

Embase 547

Cochrane 41

After endnote x9 deduplication = 801

After Rayyan deduplication = 739

***Abbreviations Key***

**PCN = Percutaneous Nephrostomy**

**Supplementary File 2: Full table of survival by cancer type**

| Malignant primary | Survival in months (weighted average of medians) | Total number of patients studied for survival by this cancer type | Number of studies giving survival data for this cancer type |
| --- | --- | --- | --- |
| GI | 6.77 | 323 | 3 |
| Prostate | 18.8 | 170 | 7 |
| Bladder | 11.7 | 144 | 7 |
| Genitourinary | 9.5 | 132 | 2 |
| Cervical | 15.9 | 117 | 6 |
| Other | 6.3 | 107 | 6 |
| Colo | 7.7 | 91 | 5 |
| Gynae | 18.4 | 39 | 3 |
| Gastric | 4.2 | 37 | 2 |
| Ovarian | 7.5 | 27 | 4 |
| Rectum | 7.1 | 23 | 2 |
| Lymphoma | 7.0 | 15 | 3 |
| Uterine-cervix | 14.1 | 15 | 1 |
| UGI | 4.9 | 13 | 1 |
| LGI | 8.4 | 10 | 1 |
| Breast | 10.6 | 10 | 3 |
| Sarcoma | 14.9 | 9 | 1 |
| Gallbladder | 0.2 | 1 | 1 |

***Abbreviations Key***

**GI = Gastrointestinal**

**UGI = Upper gastrointestinal**

**LGI = Lower gastrointestinal**

**Supplementary File 3: Table of included papers**

| Author | Year of publication | Type of Trial | Single centre / Multicentre | Intervention to manage obstruction | N = | Malignancy type | Metastatic status of patients |
| --- | --- | --- | --- | --- | --- | --- | --- |
| Alawneh^[41](#_ENREF_41)^ | 2016 | Retrospective | Single | PCN | 211 | Gastrointestinal, Genitourinary, Lymphoma, metastatic, not specified | Not clearly defined |
| Alma[^28^](#_ENREF_28) | 2020 | Retrospective | Single | PCN | 147 | Bladder, Prostate, Colon, Cervix, Rectum, Uterus, Stomach | Not clearly defined |
| Asakawa[^47^](#_ENREF_47) | 2018 | Retrospective | Single | Stent - metal | 52 | Gastric, Colorectal, Gynae, Breast, Pancreas, Lung, Unknown, Urogenital | Not clearly defined |
| Asakawa[^48^](#_ENREF_48) | 2018 | Retrospective | Single | Stent - Metal or Polymer | 92 | Gastrointestinal, Gynaecological, Urogenital, other | Not clearly defined |
| Azuma[^49^](#_ENREF_49) | 2013 | Retrospective | Single | PCN | 214 | Gastric, Colorectal, Uterine, Ovarian, Urothelial, Prostate, Lung, Pancreatic, Breast, other | Not clearly defined |
| Barton[^50^](#_ENREF_50) | 1992 | Retrospective | Single | PCN followed by metallic stent | 9 | Cervical | Not clearly defined |
| Bordinazzo^[51](#_ENREF_51)^ | 1994 | Retrospective | Single | PCN | 28 | Prostate | Not clearly defined |
| Botkin[^23^](#_ENREF_23) | 2021 | Retrospective | Single | PCN or stent | 179 | Cervical | Not clearly defined |
| Brin[^52^](#_ENREF_52) | 1975 | Retrospective | Single | PCN | 47 | Bladder, Rectosigmoid, Prostate, Cervix, Ovary, Breast, Lymphosarcoma, Ureter, Unkown | Metastatic and non-metastatic |
| Chapman[^53^](#_ENREF_53) | 1991 | Retrospective | Single | PCN | 17 | Bladder, Cervical, Colon, Ovary, Bronchus, Oesophageal, Prostate, Breast | Metastatic and non-metastatic |
| Chefchaouni^[54](#_ENREF_54)^ | 1998 | Prospective | Single | PCN | 31 | Prostate | Not clearly defined |
| Chen[^34^](#_ENREF_34) | 2019 | Retrospective | Multi-centre | Stent - metal | 76 | Cervical , Ovarian, Rectal, Bladder, Prostate, PMT, Gastric, MRT | Not clearly defined |
| De Souza[^55^](#_ENREF_55) | 2016 | Retrospective | Single | PCN | 45 | Cervical | Not clearly defined |
| Donat^[56](#_ENREF_56)^ | 1996 | Retrospective | Single | Stent (double J polymer) +/- PCN | 78 | Colorectal, Cervical, Sarcoma, Gastric/Pancreatic, Lymphoma, Ovarian, other | Not clearly defined |
| Dowling[^29^](#_ENREF_29) | 1991 | Retrospective | Single | PCN | 22 | Prostate | Metastatic and non-metastatic |
| Ekici^[57](#_ENREF_57)^ | 2001 | Retrospective | Single | PCN | 23 | Bladder | Not clearly defined |
| Elsamra^[58](#_ENREF_58)^ | 2013 | Retrospective | Single | Stent - polymer | 34 | Bladder, Colon, Ovarian, Prostate, Endometrial, Lymphoma, Uterine, Oesophageal, Gastric, Vaginal, Cervical | Not clearly defined |
| Fallon[^33^](#_ENREF_33) | 1980 | Retrospective | Single | PCN | 100 | Prostate, Bladder, Cervix, Ovary, Vulva, Colon, Pancreas, Lymphoma, Neuroblastoma | Not clearly defined |
| Fei[^24^](#_ENREF_24) | 2012 | Retrospective | Single | Stent (double J polymer) +/- PCN | 75 | Cervical, Endometrial, Ovarian, Uterine, Vaginal, Choreocarcinoma | Metastatic and non-metastatic |
| Feuer[^59^](#_ENREF_59) | 1991 | Retrospective | Single | PCN or no intervention | 22 | Cervix, Vagina, Endometrial, Ovarian | Not clearly defined |
| Gadducci^[60](#_ENREF_60)^ | 1994 | Retrospective | Single | PCN | 10 | Cervical | Not clearly defined |
| Gunawan[^61^](#_ENREF_61) | 2022 | Retrospective | Single | PCN | 29 | Prostate, Bladder, Cervical, Ovarian, Rectal, Gastric, Vaginal, other Gynaecological, Lung | Metastatic and non-metastatic |
| Harrington[^22^](#_ENREF_22) | 1995 | Prospective | Single | PCN or Stent (double J) | 42 | Bladder, Prostate, Cervix, Colon, Breast, Lymphoma, Kidney, unknown | Not clearly defined |
| Harris[^62^](#_ENREF_62) | 2006 | Retrospective | Single | PCN | 26 | Prostate | Not clearly defined |
| Hepperlen^[37](#_ENREF_37)^ | 1979 | Retrospective | Single | Stent - pigtail | 20 | Prostate, Colon, Cervix, Bladder, Uterus | Not clearly defined |
| Hoe[^38^](#_ENREF_38) | 1993 | Retrospective | Single | PCN | 22 | Colon, Cervix, Ovary, Bladder, Prostate, Stomach. Pancreas, Pelvic seminoma | Metastatic and non-metastatic |
| Izumi[^63^](#_ENREF_63) | 2011 | Retrospective | Single | Stent - double J stent | 61 | Cervix, Endometrial, Ovarian, Gastric, Oesophageal, Rectal, Colon, Retroperitoneal, Prostate, Bladder, Ureteral Adrenal, Lung, Pancreas, Breast, Liver, Malignant Melanoma | Not clearly defined |
| Jalbani^[64](#_ENREF_64)^ | 1988 | Retrospective | Single | PCN | 40 | Bladder, Prostate, Uterine Cervix, Ovary, Rectum, L-Lymphoma, Breast, Gall Bladder | Not clearly defined |
| Javanmard^[4](#_ENREF_4)^ | 2017 | Randomised Control Trial | Single | PCN or ureteral reimplantation | 86 | Prostate | Metastatic and non-metastatic |
| Jeong[^65^](#_ENREF_65) | 2007 | Retrospective | Single | Stent - double J stent | 86 | Gastric, Rectal, Colon, Cervical, Lung, Ovarian, Breast, Cholangiocarcinoma, Gallbladder, Pancreatic, Oesophageal, Endometrial, Caecal | Metastatic and non-metastatic |
| Jiang[^9^](#_ENREF_9) | 2016 | Retrospective | Single | PCN or urinary diversion or Haemodialysis | 68 | Cervical | Not clearly defined |
| Kamiyama^[66](#_ENREF_66)^ | 2011 | Retrospective | Single | Stent +/- PCN | 53 | Gastrointestinal, Prostate, Gynaecological, Malignant Lymphoma, Breast, Gastrointestinal stromal | Metastatic and non-metastatic |
| Kanou^[67](#_ENREF_67)^ | 2007 | Retrospective | Single | Stent +/- PCN | 75 | Uterine, Cervical, Rectal, Prostate, Gastric, Bladder, Ovarian, Uterine, Retroperitoneal, Malignant Lymphoma | Not clearly defined |
| Kehoe[^68^](#_ENREF_68) | 1993 | Retrospective | Single | PCN | 7 | Cervical | Not clearly defined |
| Keidan^[69](#_ENREF_69)^ | 1988 | Retrospective | Single | PCN | 20 | Colon, Bladder, Rectum, Ovary, Cervix, Anus, Breast, Stomach, Prostate | Not clearly defined |
| Kim[^5^](#_ENREF_5) | 2018 | Randomised Controlled trial | Single | Stent - metal or double J stents | 19 | Gastric, Colorectal, Gynaecological | Not clearly defined |
| Kim[^70^](#_ENREF_70) | 2017 | Retrospective | Single | Stent +/- PCN | 284 | Gynaecological, Lung, Head and neck, Osteologic, Breast, Colorectal, Hepatobiliary, Gastric, Haematological, others | Not clearly defined |
| Kohler[^71^](#_ENREF_71) | 1980 | Retrospective | Single | PCN | 20 | Cervix, Uterus, Bladder, Prostate | Not clearly defined |
| Lapitan^[36](#_ENREF_36)^ | 2011 | Prospective | Single | PCN or stent or no intervention | 198 | Cervical | Not clearly defined |
| Lau[^72^](#_ENREF_72) | 1995 | Retrospective | Single | PCN | 77 | Cervix, Bladder, Prostate, Lymphoma, Breast, others | Metastatic and non-metastatic |
| Lee[^73^](#_ENREF_73) | 1994 | Retrospective | Single | PCN or Stent | 52 | Cervical | Not clearly defined |
| Liaw^[12](#_ENREF_12)^ | 1997 | Retrospective | Single | PCN or Stent - double J stent | 17 | Gastric adenocarcinoma | Not clearly defined |
| Lienert^[74](#_ENREF_74)^ | 2009 | Retrospective | Single | PCN | 49 | Prostate, Bladder, Colorectal, Cervical, Ovarian, Sarcoma, Pancreatic, and Breast | Metastatic and non-metastatic |
| Little[^75^](#_ENREF_75) | 2003 | Retrospective | Single | PCN +/- stent | 31 | Bladder, Prostate, Colorectal, Gynaecological | Not clearly defined |
| Liu[^76^](#_ENREF_76) | 2019 | Retrospective | Single | PCN +/- stent | 25 | Cervical, Rectal, Colon, Prostatic, Stomach, Recto-sigmoid, Endometrial, Liposarcoma, unidentified | Metastatic and non-metastatic |
| Lopez-Martinez[^77^](#_ENREF_77) | 1997 | Retrospective | Single | Stent - metal | 8 | Prostate adenocarcinoma | Metastatic only |
| Lugmayr^[78](#_ENREF_78)^ | 1996 | Retrospective | Single | Stent - self-expandable | 40 | Melanoma, Colon, Ovary, Uterus, Prostate, Breast, Mesothelioma, Lymphoma, Bladder | Not clearly defined |
| Markowitz[^79^](#_ENREF_79) | 1989 | Retrospective | Single | PCN +/- stent | 71 | Prostate, Bladder, Breast, Ovary, Colorectal, Cervix others, | Not clearly defined |
| Mason[^6^](#_ENREF_6) | 2022 | Retrospective | Multi-centre | PCN or stent or no intervention | 126 | Gynaecological | Metastatic and non-metastatic |
| Matsuura[^80^](#_ENREF_80) | 2020 | Retrospective | Single | Stent - double J stent | 93 | Ovary, Endometrium, Cervix, Vagina, Sarcoma, Gastric, Colon, Rectum, Duodenum, Gallbladder, Oesophagus, Pancreas, Prostate, Lung, Peritoneum, Lymphoma, Breast, Retroperitoneal Sarcoma, unknown | Not clearly defined |
| McNamara[^81^](#_ENREF_81) | 1980 | Retrospective | Single | PCN | 11 | Lymphosarcoma, Colon, Breast, Rectum, Cervix, Non-Hodgkins Lymphoma, Testicular | Not clearly defined |
| Misra[^82^](#_ENREF_82) | 2013 | Retrospective | Single | PCN or stent | 22 | Prostate, Bladder, Gynaecological, Rectal | Metastatic and non-metastatic |
| Miyazaki[^35^](#_ENREF_35) | 2019 | Prospective | Multi-centre | Stent - metal | 46 | Gastrointestinal, Gynaecological, other | Metastatic and non-metastatic |
| Nariculam^[83](#_ENREF_83)^ | 2009 | Retrospective | Single | PCN | 25 | Prostate | Metastatic and non-metastatic |
| Noegroho^[84](#_ENREF_84)^ | 2021 | Retrospective | Single | PCN | 163 | Cervical | Metastatic and non-metastatic |
| Perri[^85^](#_ENREF_85) | 2019 | Retrospective | Single | PCN | 74 | Uterine Cervix, Ovary, Uterus | Metastatic and non-metastatic |
| Ishioka[^40^](#_ENREF_40) | 2008 | Retrospective | Single | PCN | 140 | Gastric, Colorectal, Uterine cervical, Ovarian, Urothelial, Breast, Others | Metastatic and non-metastatic |
| Pickersgill^[86](#_ENREF_86)^ | 2022 | Retrospective | Single | Stent | 78 | Colorectal, Prostate, Uterine, Ovarian, Cervical, Breast, Appendiceal, Testicular, Gallbladder, Pancreatic, Gastric, Leiomyosarcoma, Leukemia, Lung, Unspecified squamous cell, | Not clearly defined |
| Plesinac^[13](#_ENREF_13)^ | 2010 | Retrospective | Single | PCN | 117 | Cervical, Uterine, Ovarian | Metastatic and non-metastatic |
| Radecka^[87](#_ENREF_87)^ | 2006 | Retrospective | Single | PCN | 151 | Prostate, Urinary bladder, Gynaecological, Colorectal, Ureteral, Kidney, Lymphoma, Pancreas, Retroperitoneal, Gallbladder, Carcinoid, Gastric, unknown origin | Not clearly defined |
| Romero[^88^](#_ENREF_88) | 2005 | Retrospective | Single | PCN | 43 | Uterine Cervix, Bladder, Prostate, Ovary, Vulva | Not clearly defined |
| Salunkhe^[14](#_ENREF_14)^ | 2020 | Retrospective | Single | PCN +/- stenting | 52 | Cervical | Metastatic and non-metastatic |
| Seo^[21](#_ENREF_21)^ | 2017 | Retrospective | Single | PCN or stent | 284 | Gynaecological, Lung, Head and neck, Osteologic, Breast, Colorectal, Hepatobiliary, Stomach, Haematologic, others | Not clearly defined |
| Sharer[^89^](#_ENREF_89) | 1978 | Retrospective | Single | PCN or cutaneous ureterostomy or transureteroureterostomy and cutaneous ureterostomy or ileal conduit or intubated ureterostomy or ureteral catheter or stent gibbons or lysis of adhesions | 62 | Bladder, Cervix, Prostate, Gastrointestinal, Breast, Lymph, ovary, Endometrial, Choriocarcinoma, Pancreas, undifferentiated | Metastatic and non-metastatic |
| Siamplis^[90](#_ENREF_90)^ | 1996 | Retrospective | Single | PCN | 34 | Urinary bladder, Cervical, Colon, Ovarian, Prostate, | Metastatic and non-metastatic |
| Sood[^91^](#_ENREF_91) | 2006 | Prospective | Single | PCN | 32 | Bladder, Cervix, Endometrium, Rectum, Ovarian, Renal | Not clearly defined |
| Spencer[^7^](#_ENREF_7) | 2013 | Retrospective | multi-centre | PCN or Stent | 2958 | Prostate | Not clearly defined |
| Tan[^92^](#_ENREF_92) | 2019 | Retrospective | Single | PCN or stent | 89 | Cervical | Metastatic and non-metastatic |
| Tanaka[^93^](#_ENREF_93) | 2004 | Retrospective | Single | PCN | 33 | Gastric, Colorectal, Pancreas, Cholangiocarcinoma, Ovary, Cervical, Corpus, Unknown of gynaecological origin, Upper urinary tract, Prostate, Lung | Metastatic and non-metastatic |
| Tatenuma^[94](#_ENREF_94)^ | 2020 | Retrospective | Single | PCN or Stent or No intervention | 151 | Gastrointestinal, Gynaecological, other | Not clearly defined |
| Teenan [^95^](#_ENREF_95) | 1989 | Retrospective | Single | PCN | 16 | Bladder, Prostate, Cervix, Colorectal, Lymphoma | Not clearly defined |
| Tekin^[96](#_ENREF_96)^ | 2001 | Retrospective | Single | Stent - metal | 8 | Rectal, Cervical, Bladder, Prostate | Metastatic and non-metastatic |
| Tlili^[97](#_ENREF_97)^ | 2021 | Retrospective | Single | Stent - double J | 174 | Bladder, Uterine, Prostate, Colorectal, Retroperitoneal tumour | Not clearly defined |
| Tsauo^[98](#_ENREF_98)^ | 2022 | Retrospective | Single | Stent - Urexel | 129 | Gastric, Colorectal, Breast, Pancreatic, Lung, Small intestine, Bladder, miscellaneous, Peritoneal carinomatosis | Not clearly defined |
| Vogt[^99^](#_ENREF_99) | 2018 | Retrospective | Single | PCN or stent or no intervention treated or ileal conduit diversion or nephrectomy | 156 | Prostate, Bladder, Cervical, Colon, Rectal, Ovary, Pelvic, Breast, Retroperitoneal mass, Gastric, Oesophageal, Lung | Metastatic and non-metastatic |
| Watkinson[^100^](#_ENREF_100) | 1992 | Retrospective | Single | PCN | 50 | Bladder, Cervix, Colon, Lymphoma, Ovary, Synovial sarcoma, Teratoma, Lung, Chondrosarcoma, Carcinoma of unknown origin | Metastatic and non-metastatic |
| Wijayarathna^[101](#_ENREF_101)^ | 2014 | Prospective | Single | Stent or surgical diversion | 82 | Cervix, Endometrium, Rectum/Sigmoid, Retroperitoneal mass, Ovary, Prostate, Bladder, Sarcoma of pelvis, Vagina | Not clearly defined |
| Wilson[^39^](#_ENREF_39) | 2005 | Retrospective | Single | PCN | 32 | Prostate, Bladder, Colorectal, Gynaecological, Breast | Not clearly defined |
| Wong[^102^](#_ENREF_102) | 1989 | Retrospective | Single | PCN or Stent - double J | 10 | Rectum, Cervix, Stomach | Metastatic only |
| Wong[^27^](#_ENREF_27) | 2007 | Retrospective | Single | PCN or Stent | 102 | Gastrointestinal, Gynaecological, Urological, Lymphoma, Breast, Sarcoma, Lung, Melanoma, Hemangioperictyoma, unknown primary, Mucinous appendiceal carcinoma | Metastatic and non-metastatic |
| Yoon[^103^](#_ENREF_103) | 2018 | Retrospective | Single | Stent | 117 | Uterus, Ovarian, Colon, upper Gastrointestinal, Cholangiocarcinoma, Bladder, Breast | Not clearly defined |
| Zadra[^104^](#_ENREF_104) | 1987 | Retrospective | Single | PCN or stent or surgical diversion | 98 | Cervix, Prostate, Bladder, Ovary, Gastrointestinal, Breast, Lymphoma, Testis, Lung, Uterus | Metastatic and non-metastatic |

***Abbreviations Key***

**PCN = Percutaneous Nephrostomy**
